# Osmotin-derived 9-amino-acid peptide alleviates α-synuclein and MPTP-induced glial cell activation mediated neuroinflammation, protecting dopaminergic neurons in Parkinson’s disease mice brain

**DOI:** 10.1186/s12929-026-01215-4

**Published:** 2026-01-26

**Authors:** Kyonghwan Choe, Muhammad Tahir, Min Hwa Kang, Hyun Young Park, Riaz Ahmad, Tae Ju Park, Myeong Ok Kim

**Affiliations:** 1 Division of Life Science and Applied Life Science (BK21 FOUR), College of Natural Sciences, Gyeongsang National University, Jinju-si, Republic of Korea; 2 Department of Psychiatry and Neuropsychology, School for Mental Health and Neuroscience (MHeNs), Maastricht University, Maastricht, Netherlands; 3 Department of Pediatrics, Maastricht University Medical Center (MUMC+), 6202 AZ Maastricht, The Netherlands; 4 Department of Cell Biology, Albert Einstein College of Medicine, 1300 Morris Park Avenue, Bronx, NY 10461 USA; 5 Alz-Dementia Korea Co., Jinju-si, Republic of Korea

**Keywords:** Peptide osmotin-derived 9-amino-acid (Os_9aa), Parkinson’s disease (PD), Dopaminergic neuron, α-synuclein (α-syn), 1-methyl-4-phenyl-1,2,3,6-tetrahydropyridine (MPTP), Neuroinflammation, Oxidative stress

## Abstract

**Background:**

Parkinson’s disease (PD) is the second most common neurodegenerative disorder, categorized by the loss of dopaminergic neurons in the brain’s Substantia Nigra pars compacta (SNpc) due to α-synuclein (α-syn) aggregation, leading to reduced dopamine levels in the striatum. This research study evaluates the neuroprotective potential of the novel peptide osmotin-derived 9-amino-acid (Os_9aa, C-T-Q-G-P-C-G-P-T) against α-syn (neuron-specific enolase promoter human alpha-synuclein (NSE-hαSyn)) and 1-methyl-4-phenyl-1,2,3,6-tetrahydropyridine (MPTP)-induced PD models.

**Methods:**

Human neuroblastoma SH-SY5Y cells were employed as an in vitro model, while NSE-hαSyn (α-synuclein) transgenic mice and MPTP-treated mice were used as in vivo models of PD. MPTP was administered intraperitoneally (30 mg/kg) once daily for five consecutive days. Mice were immunized with Os_9aa (15 mg/kg, i.p., twice weekly for five weeks), followed by behavioral assessments including open field test, wire hang test, pole test, and rotarod test, and biochemical analysis using the Triplex Assay, western blotting, and confocal microscopy.

**Results:**

Our study demonstrated that the novel peptide Os_9aa enhanced cell viability, reduced cytotoxicity, and apoptosis in SH-SY5Y neuroblastoma cells. Os_9aa attenuated synucleinopathy-related pathology in NSE-hαSyn transgenic mice and MPTP-induced PD mouse models. Current findings also highlighted the therapeutic potential of Os_9aa in mitigating behavioral deficits observed in NSE-hαSyn and MPTP mouse models of PD. Furthermore, Os_9aa administration effectively restored key dopaminergic markers, including tyrosine hydroxylase (TH), vesicular monoamine transporter 2 (VMAT2), and dopamine transporter (DAT). Additionally, it reduced neuroinflammation by decreasing the activation of glial cells—ionized calcium-binding adaptor molecule 1 (Iba-1) and glial fibrillary acidic protein (GFAP), as well as pro-inflammatory cytokines, such as phosphorylated nuclear factor-κB (p-NF-кB), tumor necrosis factor-α (TNF-α), and interleukin-1β (IL-1β), in the striatum and SNpc regions. Furthermore, Os_9aa mitigated oxidative stress (OS) by upregulating the expression of nuclear factor erythroid-related factor 2 (Nrf-2) and heme oxygenase 1 (HO-1), and improved cognitive performance.

**Conclusion:**

Collectively, these findings highlight the neuroprotective potential of the Os_9aa, which counteracts α-synuclein– and MPTP-induced neurotoxicity by reducing oxidative stress, glial activation, and neuroinflammation. This multifaceted protection preserves neuronal integrity in both the NSE-hαSyn transgenic and MPTP-induced PD mouse models, underscoring Os_9aa as a promising therapeutic candidate for modifying PD pathogenesis.

## Introduction

Neurodegenerative diseases (NDDs) represent a diverse group of neurological disorders that are characterized by the progressive loss of neurons in the central or peripheral nervous systems [1]. Among them, Parkinson’s disease (PD) affects 6–7 million people worldwide and is marked by the degeneration of dopaminergic neurons in the SNpc, leading to reduced striatal dopamine levels. Hallmark pathological features include α-synuclein (α-syn) aggregation, neuroinflammation, oxidative stress (OS), and mitochondrial dysfunction [2, 3]. These pathological changes contribute to the manifestation of motor symptoms such as tremors, bradykinesia, and postural instability, as well as non-motor symptoms including psychiatric disturbances, sleep disorders, depression, and cognitive impairment [4–6]. Although several therapeutic approaches such as α-syn-targeted immunotherapies [7, 8], gene therapy including gene therapy vector AAV2-mediated delivery of glial cell-derived neurotrophic factor (GDNF) or neurturin) [9], and neuroprotective agents like inosine or coenzyme Q10 have shown promise in preclinical or early clinical trials [10, 11], none have consistently shown efficacy in slowing or halting disease progression. Limitations include inadequate blood–brain barrier penetration, insufficient target engagement, and variability in patient response [12, 13]. Despite ongoing research, no disease-modifying therapies exist that effectively halt the progression of PD [14].

Commonly used experimental models of PD encompass neurotoxin-induced paradigms, such as those utilizing 1-methyl-4-phenyl-1,2,3,6-tetrahydropyridine (MPTP), as well as α-syn–based models that replicate the pathogenic aggregation and propagation of the protein observed in PD [15, 16]. The first PD model used in this study involves transgenic mice that overexpress human wild-type α-syn in the SNpc and striatum [17]. Under pathological conditions, this α-syn synaptic protein can be released from neurons and propagate throughout the nervous system via both cell-autonomous and non-cell-autonomous mechanisms. Extensive research studies demonstrated that extracellular α-syn activates microglia and initiates an inflammatory response, causing neuroinflammation, thereby contributing to the PD progression [18, 19]. Another PD model is MPTP, which converts in the brain to its toxic ionic form, 1-methyl-4-phenylpyridinium ion (MPP^+^). It crosses the blood–brain barrier (BBB), inhibits complex I of the mitochondrial electron transport chain, disrupts adenosine triphosphate (ATP) production, triggers OS, and leads to dopaminergic neuronal loss [20].

Neuroinflammation is a defensive physiological response in the brain that shields the central nervous system (CNS) from internal and external factors. It is progressively recognized as a key feature in PD development [21]. Neuroinflammation is driven by glial cells, including microglia and astrocytes, which are essential for neuronal homeostasis [22]. Several studies have reported that α-syn and MPTP induce motor dysfunctions and dopaminergic neurodegeneration by altering the expression of tyrosine hydroxylase (TH), vesicular monoamine transporter 2 (VMAT2), and dopamine transporter (DAT) in the mouse brain [23–25]. Besides this, the administration of α-syn and MPTP in mice activates glial cells [26, 27] and activates p-NF-κB (phosphorylated Nuclear Factor kappa-light-chain-enhancer of activated B cells) which is a transcription factor involved in regulating immune and inflammatory responses, as well as cell survival and proliferation, lead to produce other inflammatory cytokines such as tumor necrosis factor-α (TNF-α) and interleukin-1β (IL-1β) by playing a pivotal role in the development of the pro-inflammatory signaling pathway [28]. Besides this, OS is a major factor in PD neurodegeneration. Though excessive generation of reactive oxygen species leads to OS, their normal production during metabolism is vital for cellular defense [29]. The master antioxidant genes, nuclear factor erythroid-related factor 2 (Nrf-2) pathway, and its downstream targets, establish an important role in the cellular defense mechanism [30]. The α-syn oligomerization in the brain and OS induces activation of resident non-neuronal immune glial cells, which release different proinflammatory cytokines such as TNF-α and IL-1β that further lead to neuroinflammation and neurodegeneration [31].

Numerous natural and synthetic peptides are being developed as innovative therapeutic agents to treat systemic, lung, and wound infections by modulating pro- and anti-inflammatory responses [32, 33]. Consequently, there is significant interest in producing novel bioactive peptides. Small peptides have gained great interest as therapeutic development targets due to their immunomodulatory properties. Low molecular weight bioactive peptides, in particular, are increasingly considered promising candidates as therapeutic agents because of their wide-ranging activity within the innate immune system [34, 35]. In line with these studies, osmotin is an extracted protein of the tobacco plant that is the homolog of the mammalian hormone adiponectin based on its structural and functional comparison [36]. Osmotin is believed to have biological applications as an adiponectin agonist and was shown to reduce the anti-inflammatory effect of adiponectin in a murine colitis model [37]. Numerous studies have confirmed that adiponectin acts as a neuroprotective agent against different neurotoxic insults, e.g., kainic acid-induced excitotoxicity in rat hippocampal neurons and (MPP^+^)-induced apoptosis in human SH-SY5Y neuroblastoma cells [38, 39]. We, therefore, developed a novel osmotin-derived 9-amino-acid peptide (Os_9aa) with nine amino acids sequence (C-T-Q-G-P–C-G-P–T) and explored its therapeutic potential against NSE-hαSyn/MPTP-induced PD mouse models. The key objectives of the current study were to investigate glial activation, neuroinflammation, OS, cytotoxicity, neuronal apoptosis, and the neuroprotective potential of Os_9aa in NSE-hαSyn/MPTP-induced mouse PD models using behavioral experiments, biochemical assays, western blotting, and immunofluorescence. Notably, we found that Os_9aa maintained neuronal homeostasis, reduced motor dysfunction, neuronal apoptosis, OS, and neuroinflammation-mediated neurodegeneration in mice brains.

## Materials and methods

### Chemicals

MPTP was obtained from Sigma-Aldrich (St. Louis, MO, USA). Os_9aa was purchased from Peptron (Yuseong-daero, Daejeon, South Korea).

### Animal groupings and drug treatment

Two mice models (α-syn and MPTP) of PD are studied. C57BL/6-Tg (neuron-specific enolase promoter human alpha-synuclein (NSE-hαSyn)) Korl mice were obtained from the National Institute of Food and Drug Safety Evaluation (NIFDS, Cheongju, Korea). Animals were well-arranged in the groups (n = 8 mice/group) and were controlled as previously described [40]. Male wild-type C57BL/6N mice seven weeks old with an average body weight of 25–28 g (n = 8 mice/group) were purchased from Samtako Biolabs (Ulsan, South Korea). All mice were housed at 25 °C and acclimatized for one week under a 12 h dark/light cycle with (60 ± 10%) humidity control and provided free access to food and water. The protocols for the experimental procedures were evaluated and approved (Approval ID: 125, code: GNU-200331-M0020) by the animal ethics committee (IACUC) of the Division of Applied Life Sciences, Department of Biology at Gyeongsang National University, Republic of Korea.

**Fig. 1:**
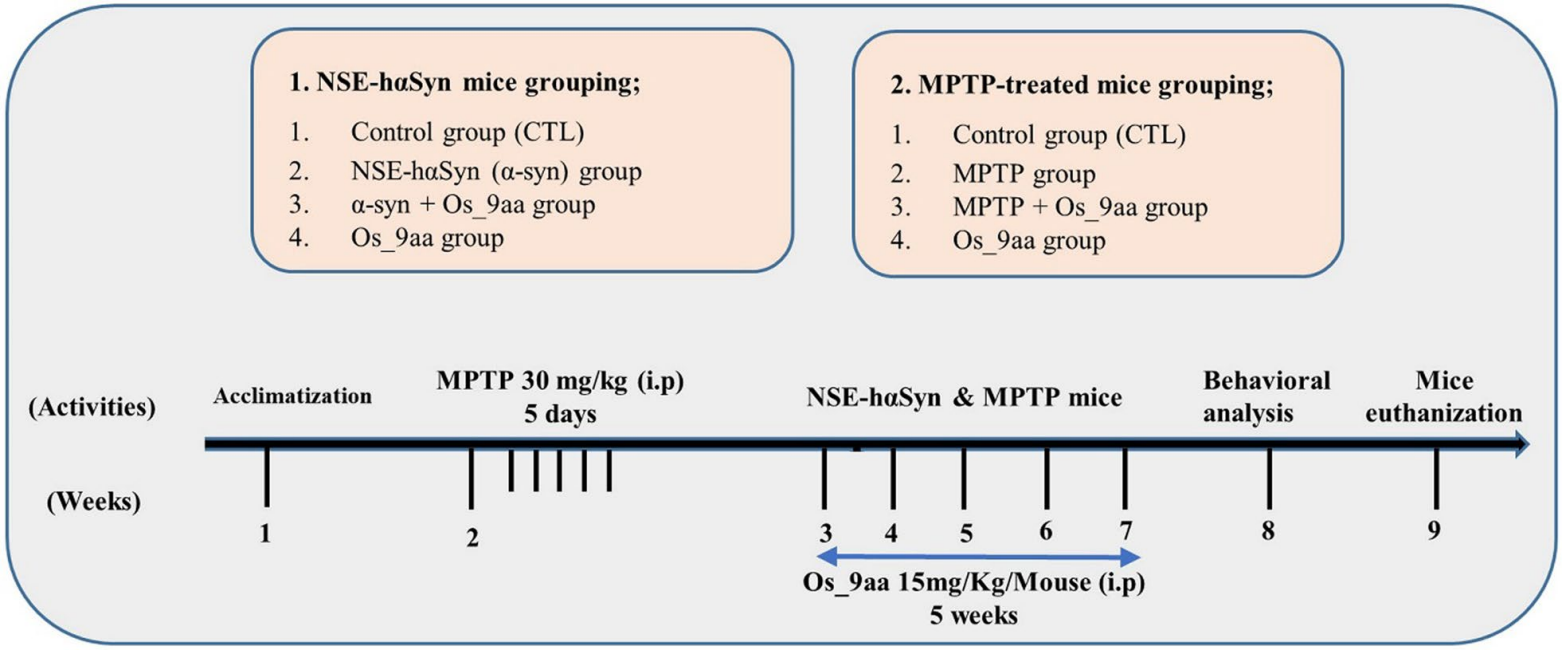
Experimental paradigm of Os_9aa against NSE-hαSyn and MPTP Parkinson’s disease mice models

To establish the dosing regimen, we optimized the administration of Os_9aa according to our preliminary studies [40]. A single intraperitoneal (i.p.) dose of 15 mg/kg Os_9aa, dissolved in 0.9% NaCl saline, was administered. Control mice received an equivalent volume of saline via (i.p.) injection. MPTP of 30 mg/kg dose was dissolved in sterile distilled water and administered via (i.p.) injection for five consecutive days [41]. MPTP mice were treated with Os_9aa via (i.p.) injections twice a week for five weeks, starting at 9 weeks of age and continuing through 14 weeks. Similarly, C57BL/6-Tg (NSE-hαSyn) Korl mice were administered Os_9aa via (i.p.) injection, following the same treatment protocol as that applied to the MPTP-treated group. The NSE-hαSyn mice were divided into the following four groups (Fig. 1): (1) control (CTL; vehicle-treated), (2) NSE-hαSyn (α-syn), (3) α-syn + Os_9aa, (4) CTL + Os_9aa. Similarly, wild-type C57BL/6N mice were divided into the following four groups: (1) control (CTL; vehicle-treated), (2) MPTP (MPTP-treated group, 30 mg/kg i.p.), (3) MPTP + Os_9aa, (4) CTL + Os_9aa.

### Behavioral tests

#### Open-field test

The evaluation of spontaneous locomotor activity was conducted using an open-field apparatus measuring (40 × 40 cm in diameter, and 40 cm in height) as described previously [42]. Before the experiments, the mice were allowed to explore the new environment for a few hours. Each mouse was individually placed in a designated corner of the arena. To eliminate any kind of smell indications, the open field was accurately cleaned with a 10% ethanol solution and allowed to dry between trials. Movement data were captured using Video tracking software 3.0 (Panlab, Barcelona, Spain) as the mice crossed the 16 equal squares within the open field. Each session continued for 5 min. The primary parameters assessed included the total distance traveled (measured in centimeters) and the duration spent in the central zone (measured in seconds).

#### Pole test

The pole used in the test is a wooden stick measuring 40 cm in length and 10 mm in diameter. Mice were familiarized with the behavioral room and underwent three training sessions per day for two consecutive days. During the test, each mouse was positioned at the top of the vertical wooden stick, oriented with its head facing upward. The time taken for the mouse to climb down the pole and place its front paws on the ground was measured and recorded as total time [43].

#### Wire hang test

The wire hang test apparatus consisted of an iron wire stretched between two poles horizontally. The mice were acclimatized in the behavioral room before the experiment. Each mouse was placed on a thin stretched wire 20 cm above the ground surface, held with its forelimbs, and the time that each mouse remained on the wire was recorded (in seconds). The test was repeated eight times for each group with the mice resting between several trials [44].

#### Rotarod test

The rotarod test apparatus (ROTA-ROD, B.S Techno lab INC., Seoul, Korea) consists of five adjacent rods, which are 440 mm in height and 345 mm in width. During the experiment, each mouse was placed on the rods, which rotated at gradually increasing speeds. The mice underwent training at 12–20 revolutions per minute (rpm). Each trial lasted for 120 s and was repeated three times for a three-day session. The duration each mouse stayed on the rod was recorded, and the average time was calculated for analysis [45].

### Cell culture

Human neuroblastoma SH-SY5Y cells, purchased from the Korea Cell Line Bank (KCLB, South Korea), were cultured in Dulbecco’s Modified Eagle Medium (DMEM; Gibco by life technologies, Grand Island, NY, USA) supplemented with 10% fetal bovine serum and 1% antibiotic–antimycotic (100 ×) Solution. Cultures were maintained at 37 °C in a humidified atmosphere with 5% CO_2_.

#### Plasmid transfection

The EGFP-alpha-synuclein-A53T plasmid was provided as a gift by David Rubinsztein (Addgene #40,823 Adiponectin receptor (AdipoR1) was knocked out using a commercial AdipoR1 CRISPR/Cas9-KO plasmid (Santa Cruz, CA, USA), and a puromycin resistance gene was inserted with an AdipoR1 HDR plasmid (Santa Cruz, CA, USA) for selection. Cells were transfected with these plasmids using Lipofectamine 3000 (Invitrogen, CA, USA) following the manufacturer’s instructions. After 24 h, the medium was replaced, and cells were selected with 2.5 µg/ml puromycin to create a stable knockout cell line [40].

#### ApoTox-Glo triplex assay

The ApoTox-Glo Triplex Assay (Promega, WI, USA) was used to assess cell viability, cytotoxicity, and caspase-3/7 activation (apoptosis) and was performed as previously described [46]. Subsequently, cells were seeded into a 96-well plates (1 × 10^5^ cells/well) and exposed to 200 μl of Dulbecco’s modified Eagle medium (DMEM) containing varying concentrations (0.5 μM, 1.0 μM, 1.5 μM, 2.0 μM, 2.5 μM) of Os_9aa along with α-syn and MPTP at 37 °C for 24 h. For the assay, 20 μl of the viability/cytotoxicity reagent containing both glycylphenylalanyl-aminofluorocoumarin (GF-AFC) and bis-alanyl-alanyl-phenylalanyl-rhodamine 110 (AAF-R110) substrates were added to all of the wells and briefly mixed using orbital shaking (500 rpm for 30 s) and incubated for 1 h at 37 °C. Cell viability was assessed fluorometrically using excitation/emission wavelengths of 400 nm/505 nm, while cytotoxicity was measured at 485 nm/520 nm. Apoptotic activity was evaluated using a luminogenic caspase-3/7 substrate following the manufacturer’s instructions. The results were calculated as % cell viability, % cytotoxicity, and % caspase-3/7 activity.

### Protein extraction

Following the behavioral analysis, the mice were anesthetized intraperitoneally (i.p.) with ketamine and xylazine and then euthanized and brain was removed [47]. The brain tissues were dissected carefully and the striatum and substantia nigra pars compacta (SNpc) regions were separated. The striatum and SNpc were individually homogenized in PRO-PREP™ protein extraction solution (iNTRON Biotechnology, Inc., Sungnam, South Korea), and centrifuged at 13,000 rpm for 25 min at 4 °C. The supernatants were collected and kept at − 80 °C for further processing.

### Immunoblot analysis

Briefly a Bio-Rad assay kit (Bio-Rad Laboratories, Irvine, CA, United States) was used to measure protein concentrations as described previously [48]. Accordingly, the proteins (20–25 mg) from the brain were electrophoresed by SDS-PAGE on 12–15% gels in comparison under reducing conditions and transferred to polyvinylidene difluoride (PVDF) membranes (Immobilon-PSQ, Transfer membrane, Merck Millipore, Burlington, MA, United States). Prestained protein ladders (Gangnam-STAIN, iNtRON Biotechnology, Seoul, South Korea) were loaded in parallel (as a molecular weight control). The membranes were blocked with 5% skim milk (Difco™ Skim Milk, BD, France) to prevent non-specific binding and further incubated with primary antibodies at 4 °C overnight (Table 1). After incubation, the membranes were washed and then probed with horseradish peroxidase (HRP)-conjugated secondary antibodies. The bands were visualized using an enhanced chemiluminescent (ECL) detection reagent (ATTO Corporation, Tokyo, Japan), and the band quantification was carried out using ImageJ software [30].

**Table 1:** List of antibodies used in western blotting and confocal microscopy

Protein	Host	Application	Manufacturer	Catalog number	Dilution
α-syn	Mouse	WB/IF	Santa Cruz Biotechnology	SC58480	1:1000/1:100
TH	Rabbit	WB/IF	Merck	AB152	1:1000/1:100
DAT	Rabbit	WB	Santa Cruz Biotechnology	SC32259	1:1000
VMAT2	Rabbit	WB	Abcam	AB70808	1:1000
GFAP	Mouse	WB/IF	Santa Cruz Biotechnology	SC33673	1:100
Iba-1	Rabbit	WB/IF	Abcam	Ab178846	1:1000/1:100
p-NF-кB	Mouse	WB	Santa Cruz Biotechnology	SC293132	1:1000
TNF-α	Mouse	WB	Santa Cruz Biotechnology	SC52746	1:1000
IL-1β	Mouse	WB	Santa Cruz Biotechnology	SC32294	1:1000
Nrf-2	Mouse	WB/IF	Santa Cruz Biotechnology	SC365949	1:1000/1:100
HO-1	Mouse	WB	Santa Cruz Biotechnology	SC136961	1:1000
β-actin	Mouse	WB	Santa Cruz Biotechnology	SC 47778	1:1000

### List of antibodies

#### Immunofluorescence staining

After behavioral analysis, mice were anesthetized and perfused transcardially with normal saline (0.9%) and 4% paraformaldehyde as described previously [30]. The brains were carefully removed and preserved in ice-cold 4% neutral buffer paraformaldehyde at 4 °C for 72 h. Subsequently, they underwent a dehydration process in 20% sucrose for another 72 h. The brain was kept frozen in an optimal cutting temperature (O.C.T) compound (Sakura Finetek USA, Inc., Torrance, CA, USA), and brain sections with 14 μm thickness were obtained on gelatin-coated slides by using a microtome (CM 3050C cryostat, Leica, Germany).

Immunofluorescence histology was conducted according to previous protocols [49]. Brain tissues were exposed to a 10-min wash with 0.01 M PBS and then treated with proteinase K for 5 min. After PBS washing, brain sections were blocked with normal serum (Vector, diluted 1:20 in PBS) for 1 h. Incubation with specific primary antibodies was carried out overnight at 4 °C. Following incubation, brain sections underwent washing with PBS and subjected to secondary antibodies (tetramethylrhodamine isothiocyanate (TRITC) or fluorescein isothiocyanate (FITC) (anti-rabbit, anti-goat, or anti-mouse)) diluted 1:50 in PBS for 90 min at room temperature. A nucleus counterstaining solution 4′,6-diamidino-2-phenylindole (DAPI) was applied to tissue slides which were further prepared with mounting media by applying DPX (Distyrene Plasticizer Xylene) and covered with glass coverslips. Immunofluorescence imaging was achieved using a confocal laser scanning microscope (FV 1000MPE, Olympus, Japan).

### Statistical analysis

The statistical data are expressed as Mean ± Standard Error Mean (S.E.M) based on four independent experiments. For analysis of variance, A one-way ANOVA (analysis of variance) followed by independent Student’s t-test and Tukey’s multiple comparison analysis were used for comparison among the different groups, and p < 0.05 was considered statistically significant. The western blot and immunofluorescence images were quantified using ImageJ software. All experimental data were analyzed using GraphPad Prism 8 (version 8.0.2).

## Results

### Os_9aa alleviates α-syn and MPTP-induced behavioral and motor dysfunction

**Fig. 2:**
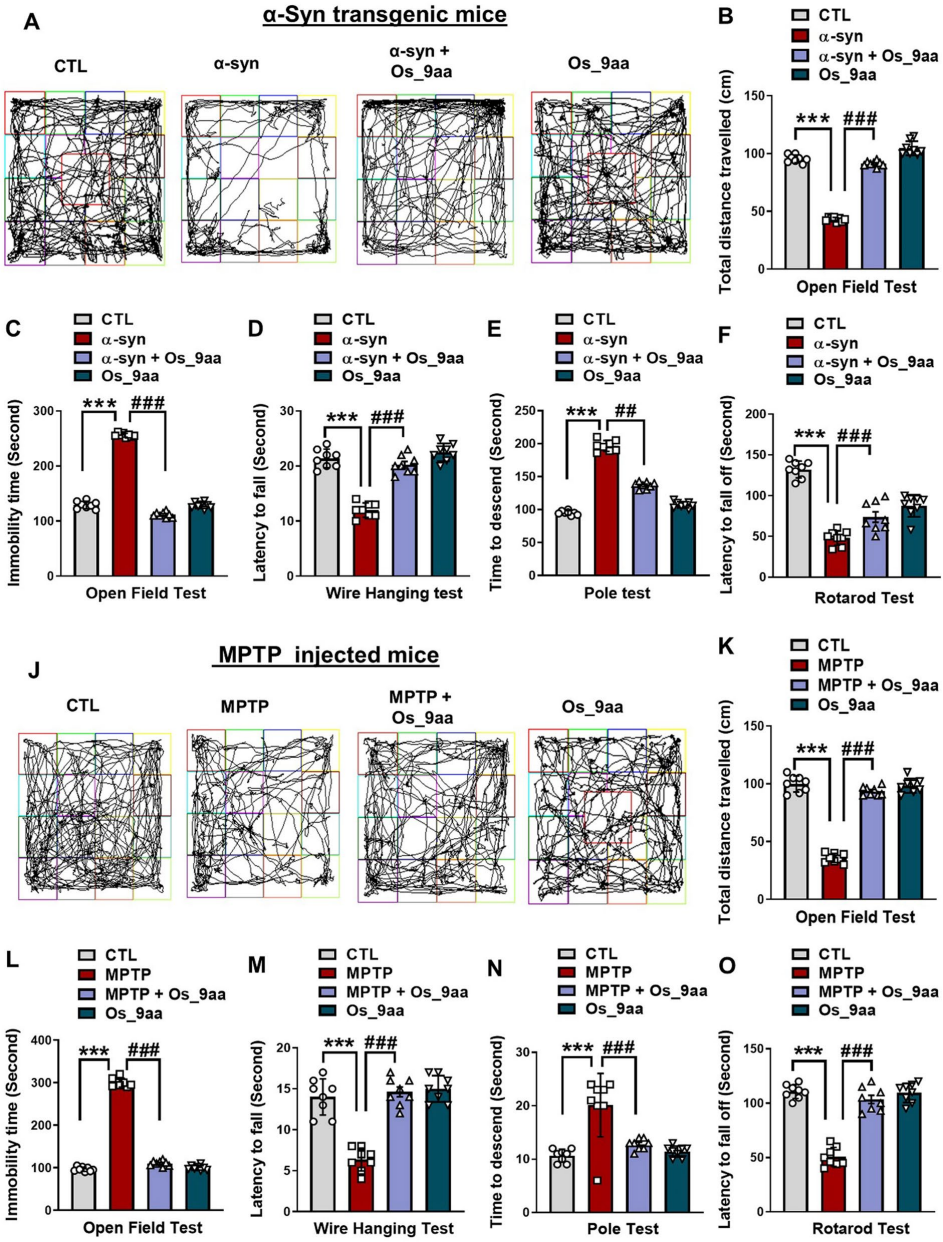
Os_9aa ameliorates motor dysfunctions in the NSE-hαSyn and MPTP mice. **A**–**F** illustrations indicate NSE-hαSyn mice and (**J–O**) represent MPTP-treated mice (n = 8 mice/group/model). **A** and **J** shows the path trajectories of mice in an open-field test. **B, C** and (**K, L**) Quantitative examination of the mice movements, the area covered in the form of total distance and immobility time spent by the mice in the open field box. **D, M** represent the latency to fall in the wire-hanging test of α-syn and MPTP mice groups. Histograms (**E, N**) depict the time to descend to the ground in the pole test. **F, O** show the latency to fall off in the rotarod test. The values in the graphs show significant differences in mean ± SEM. *Significant difference between the control and α-syn and MPTP; # difference between α-syn and MPTP and Os_9aa treated. Significance: #p ≤ 0.05, ##p ≤ 0.01, and ###p ≤ 0.001; *p ≤ 0.05, **p ≤ 0.01, and ***p ≤ 0.001

We investigated the protective effects of Os_9aa against motor deficits in NSE-hαSyn and MPTP mice as determined by various behavioral tests. In the open-field test, compared to control mice, NSE-hαSyn and MPTP mice showed a significant reduction in the total distance covered and enhanced immobility time in α-syn and MPTP-treated mice. However, these effects were significantly reversed in the Os_9aa-treated groups (Fig. 2A–C and Fig. 2J–L) respectively. Next, the effects of Os_9aa on bradykinesia and neuromuscular strength were assessed by performing a wire hang test (Fig. 2D and M). In the wire-hang test, the α-syn and MPTP groups revealed a reduced latency to fall, however, Os_9aa treatment increased the latency to fall in Os_9aa-treated mice groups. Further, a pole test was performed to assess motor coordination and bradykinesia in mice. In the pole test, compared to the control group, the α-syn and MPTP groups showed increased total time to return to the floor, but Os_9aa treatment reduced the total time (Fig. 2D and N). The rotarod test was used to assess the locomotor activity and movement control of mice evaluating latency to fall from the accelerating rod. The duration before falling from the accelerating rod was recorded. Mice of the α-syn and MPTP group remained on the rod for a shorter time, while those vaccinated with Os_9aa groups exhibited improved equilibrium and stayed on the rod significantly longer (Fig. 2F and O). Among these, the Os_9aa treated groups of mice demonstrated a higher latency to fall compared to the α-syn and MPTP mice groups [45, 50].

### Os_9aa attenuates α-syn and MPTP-induced cell viability reduction, cytotoxicity, and apoptosis in SH-SY5Y human neuroblastoma cells

**Fig. 3:**
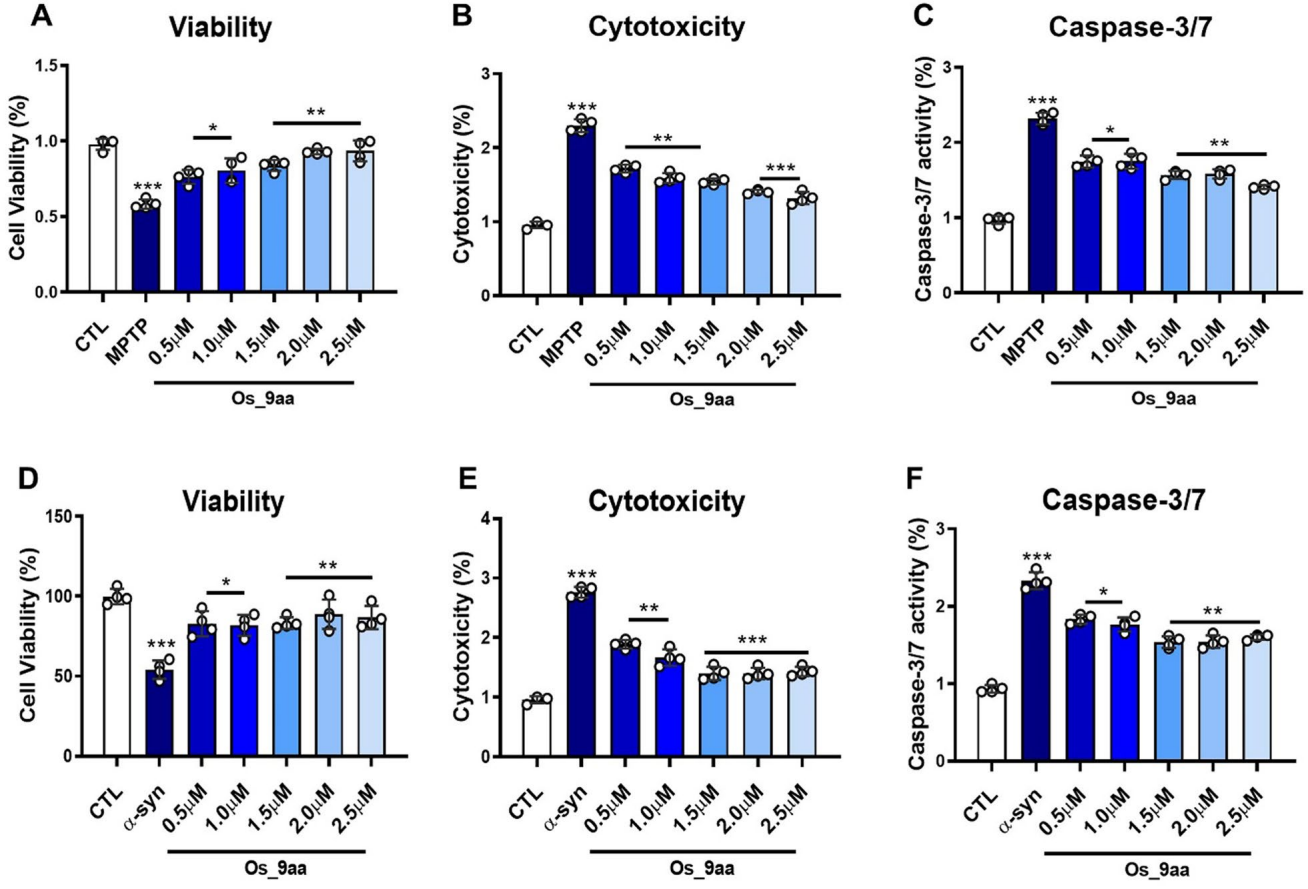
Os_9aa protects against α-syn and MPTP treated SH-SY5Y human neuroblastoma cell via in vitro Triplex assay. **A**–**C** Histograms denote Cell viability, cytotoxicity, and caspase-3/7 at different concentration of Os_9aa respectively in MPTP-treated SH-SY5Y cells. **D**–**F** denote α-synuclein (A53T)-transfected Cell viability, cytotoxicity, and caspase-3/7 at different concentration of Os_9aa respectively. The values in graphs show significant differences in mean ± SEM. *Significant difference between the control and α-syn and MPTP; # difference between α-syn and MPTP and Os_9aa treated. Significance: #p ≤ 0.05, ##p ≤ 0.01, and ###p ≤ 0.001; *p ≤ 0.05, **p ≤ 0.01, and ***p ≤ 0.001

To investigate the effects of the Os_9aa on α-syn and MPTP-induced toxicity in vitro, we first evaluated MPTP-induced cell viability, cytotoxicity, and caspase-3/7 activity at varying doses (Fig. 3A–C). We began by examining the protective effects of Os_9aa against MPTP cytotoxicity using human neuroblastoma SH-SY5Y cells, a common model for PD studies. Os_9aa was tested at five concentrations (0.5 μM, 1.0 μM, 1.5 μM, 2.0 μM, and 2.5 μM) to assess its influence on cell viability, cytotoxicity, and caspase-3/7 activity. Similarly, we evaluated the effects of Os_9aa on the A53T-induced cell lines (Fig. 3D–F). The results revealed that SH-SY5Y cells treated with Os_9aa significantly restored the cell viability in a dose-dependent manner and reduced the cytotoxic cell death and caspase-3/7 activity (Fig. 3A–C, and Fig. 3D–F).

### Os_9aa reduces α-syn accumulation and protects PD‑associated pathologies

**Fig. 4:**
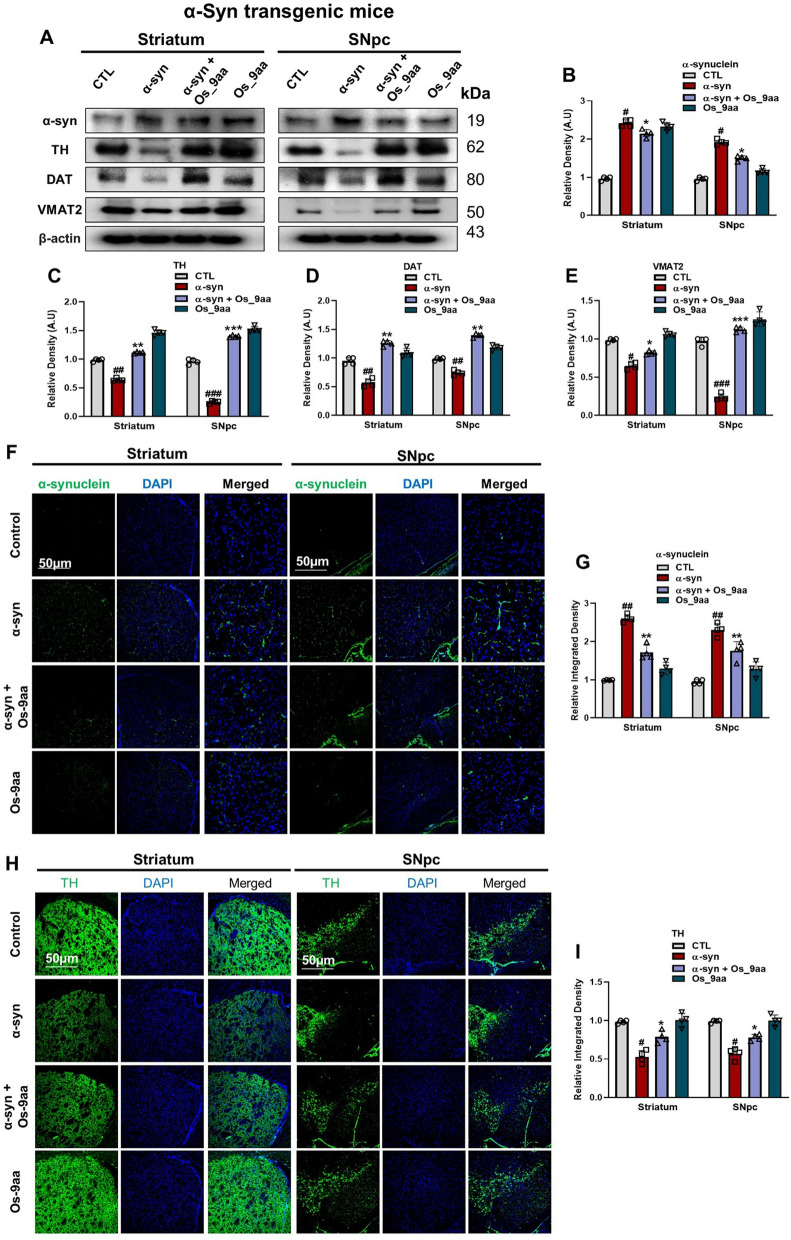
Os_9aa reduces α-syn accumulation and restore expression of dopamine-related protein in the striatum and SNpc of NSE-hαSyn mice brain. (**A**) Shows western blot analysis along with their respective histograms (**B**–**E**) of α-syn expression and dopamine-related proteins TH, DAT, and VMAT2 in the striatum and SNpc in NSE-hαSyn mice respectively, (n = 4 mice/group/model). β-actin was used as a loading control. Band quantification of all the groups was performed using ImageJ software. (**F**, **G**) Immunofluorescence reactivity of α-syn with corresponding graph in the striatum and SNpc in NSE-hαSyn mice respectively. (**H**, **I**) shows immunoreactivity of TH with respective graph in the striatum and SNpc in NSE-hαSyn mice. Magnification 10 × , scale bar = 50 µm. The values in graphs show significant differences in mean ± SEM. *Significant difference between the control and α-syn; # difference between α-syn and Os_9aa treated. Significance: #p ≤ 0.05, ##p ≤ 0.01, and ###p ≤ 0.001; *p ≤ 0.05, **p ≤ 0.01, and ***p ≤ 0.001

**Fig. 5:**
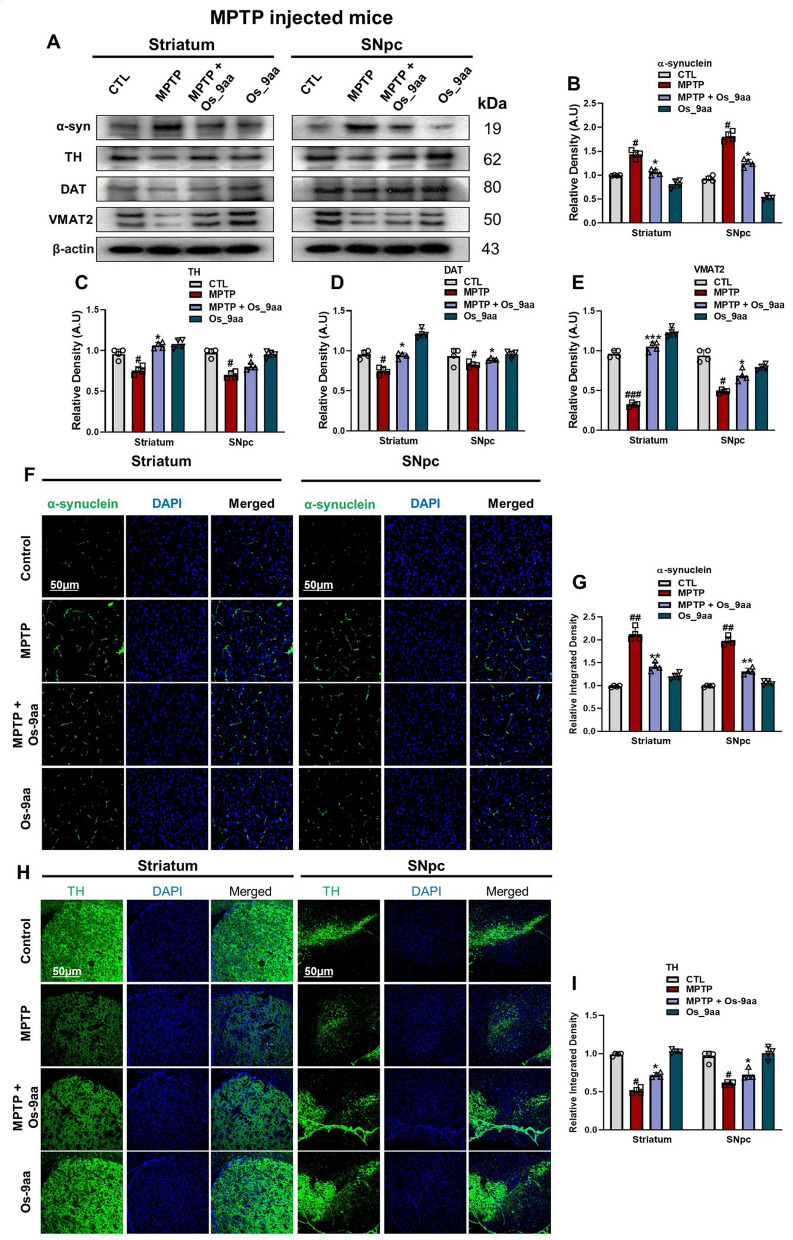
Os_9aa mitigates MPTP-induced α-syn accumulation and reinstates dopamine-related protein expression in the striatum and SNpc of MPTP-induced mice. Panel (**A**) along with associated histograms (**B**–**E**), illustrates western blot analyses of α-syn and dopamine-related proteins TH, DAT, VMAT2 in the striatum and SNpc of MPTP-treated mice (n = 4 mice per group). β-actin was used as the loading control, and band quantification for all groups was performed using ImageJ software. (**F**, **G**) present immunofluorescence reactivity for α-syn with corresponding graphs for the striatum and SNpc, while panels (**H**, **I**) depict immunoreactivity for TH with respective graphs in these regions. Images were captured at 10 × magnification, with a scale bar of 50 µm. The graphs represent significant differences as mean ± SEM. Statistical significance is indicated as follows: * denotes differences between the control and MPTP groups, and # indicates differences between the MPTP and Os_9aa -treated groups. Levels of significance are represented as *p ≤ 0.05, **p ≤ 0.01, ***p ≤ 0.001; #p ≤ 0.05, ##p ≤ 0.01, ###p ≤ 0.001

In PD pathogenesis, the degeneration of dopaminergic neurons is associated with the misfolding and aggregation of α-syn in the brain. This protein is overexpressed in PD mouse models of NSE-hαSyn and MPTP [51]. The pathogenesis of PD involves dopaminergic neuronal markers that play crucial roles in dopamine synthesis and transport, including Tyrosine Hydroxylase (TH), Vesicular Monoamine Transporter 2 (VMAT2), and Dopamine Transporter (DAT) [52]. We evaluated the effects of Os_9aa on α-syn expressions in NSE-hαSyn and MPTP mice brain, our western blot and immunofluorescences results showed that Os_9aa treatment in PD mouse models significantly reduced the expression of α-syn. in the striatum and SNpc brain regions in α-syn and MPTP mice brain compared to control mice and was restored after Os_9aa administration (Fig. 4 A–E) and (Fig. 5A–E). Os_9aa treatment significantly increased the levels of dopaminergic neuron markers, including TH, VMAT2, and DAT, in the striatum and SNpc of α-syn and MPTP mice brain. Immunofluorescence analysis also demonstrated that α-syn and TH levels, which were reduced in the α-syn and MPTP mice brain, were elevated in mice co-treated with Os_9aa. These results suggest that Os_9aa protects dopaminergic neurons from the neurotoxicity associated with PD pathology (Fig. 4F–I) and (Fig. 5F–I).

### Os_9aa mitigated Glial cell’s activation and neuroinflammation in NSE-hαSyn and MPTP mice brains

The NSE-hαSyn and MPTP mice exhibited enhanced activation of astrocytes and microglia cells [53, 54]. In the PD brain, neuronal cells release a small amount of α-synuclein protein, which is absorbed by glial cells and stimulates the expression of immune-related genes [55]. GFAP (glial fibrillary acidic protein) and Iba1 (ionized calcium-binding adaptor protein-1) are two specific markers of activated astrocytic and microglial cells in the brain, respectively [56].

**Fig. 6:**
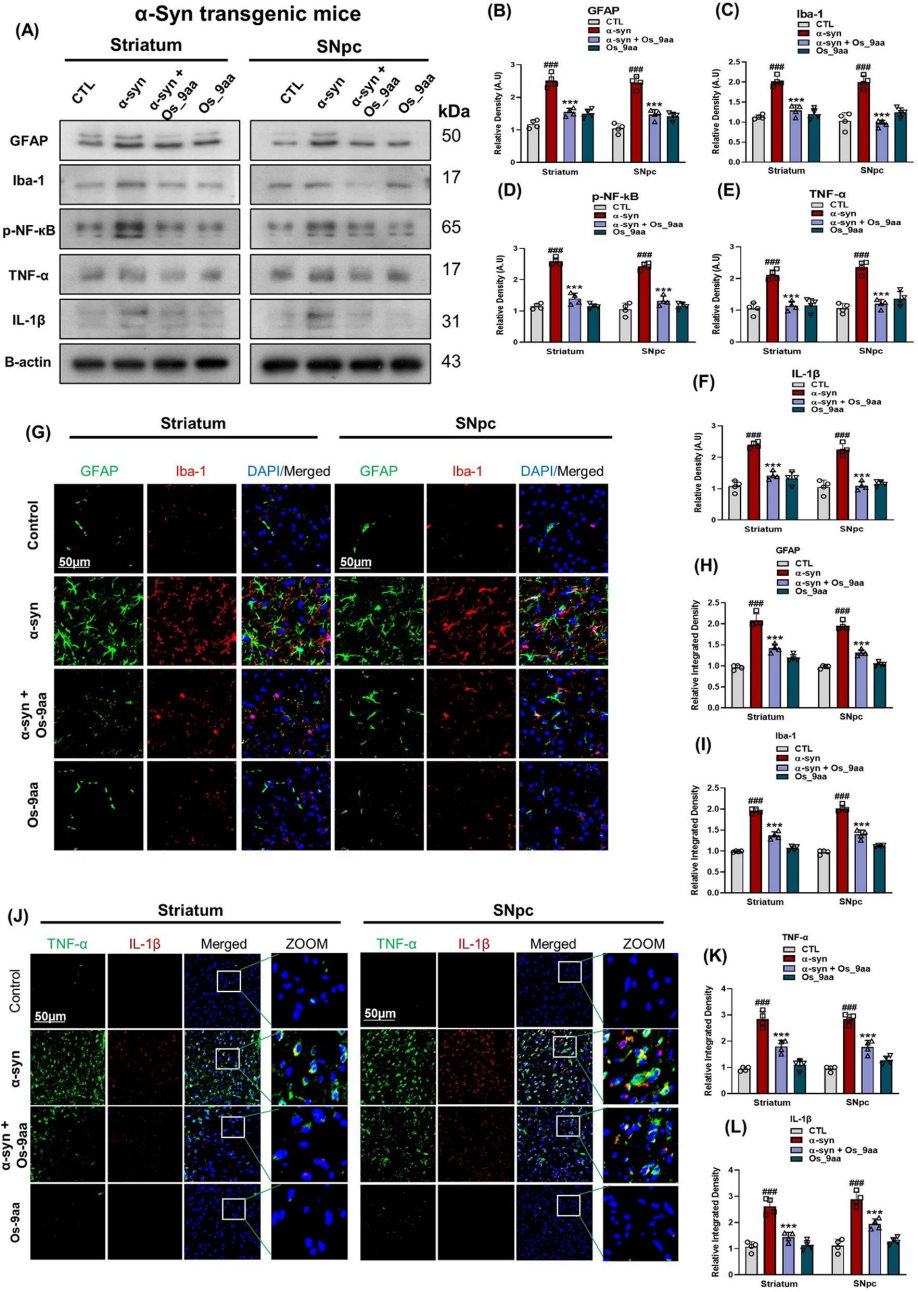
Os_9aa inhibited glial cell activation and inflammatory cytokines in NSE-hαSyn mice brains. **A** Shown western blot analysis and (**B, C, D, E, F**) graphical representations of GFAP and Iba-1 and inflammatory cytokines, p-NF-кB, TNF-α, and IL-1β in the striatum and SNpc in NSE-hαSyn mice, respectively. As a loading control, β-actin was used. (**G, H, I**) Immunoreactivity of colocalized GFAP and Iba-1 and (**J, K, L**) TNF-α and IL-1β in the brain’s striatum and SNpc respectively. Magnification 10 **×** , scale bar = 50 µm. The values in the graphs show significant differences in mean ± SEM. *Significant difference between the control and α-syn; # difference between α-syn and Os_9aa treated. Significance: #p ≤ 0.05, ##p ≤ 0.01, and ###p ≤ 0.001; *p ≤ 0.05, **p ≤ 0.01, and ***p ≤ 0.001

**Fig. 7:**
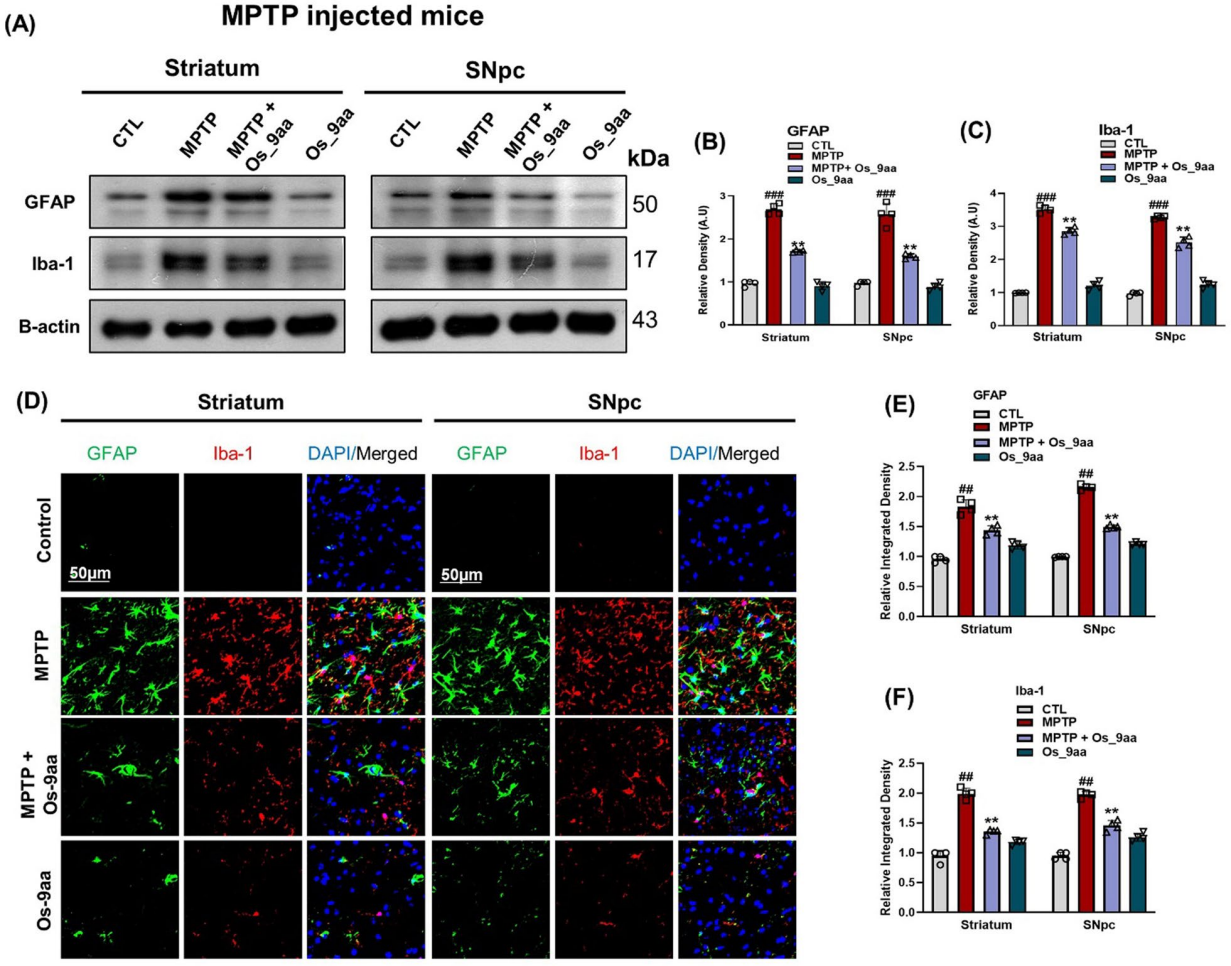
Os_9aa alleviated the MPTP-induced activation of glial cells. (**A**) Shown immunoblot analysis and their respective histograms (**B**, **C**) of GFAP and Iba-1 in the striatum and SNpc in the MPTP-treated mice respectively. β-actin as a loading control was used. (**D**) Immunofluorescence of colocalized GFAP and Iba-1 with their respective histogram (**E**, **F**) in the striatum and SNpc in the MPTP-treated mice respectively. Magnification 10 × , scale bar = 50 µm. The values in the graphs show significant differences in mean ± SEM. *Significant difference between the control and MPTP; # difference between MPTP and Os_9aa treated. Significance: #p ≤ 0.05, ##p ≤ 0.01, and ###p ≤ 0.001; *p ≤ 0.05, **p ≤ 0.01, and ***p ≤ 0.001

In current study immunoblot analysis, we studied the effects of Os_9aa against α-syn and MPTP activated glial cells, which showed higher expressions of GFAP, and Iba1 in the PD-induced mice striatum and SNpc as compared to saline-treated normal mice. At the same time, treatment with Os_9aa reduced the expression of activated glial cells. During p-NF-кB activation, which is a transcription factor, it causes to activate other inflammatory cytokines such as TNF-α and IL-1β that further lead to the development of inflammatory cascades (Fig. 6A–F). Together with this, we also investigated the expressions of different inflammatory markers such as, TNF-α and IL-1β to study anti-inflammatory effects of Os_9aa in NSE-hαSyn transgenic mice brains (Fig. 6A–F). To further validate the findings from immunoblot analyses, we employed confocal microscopy to evaluate the immunoreactivity of GFAP and Iba1 in the brains of NSE-hαSyn and MPTP mice. Additionally, we analyzed the colocalization of TNF-α and IL-1β in the brains of NSE-hαSyn transgenic mice. The immunofluorescence analysis revealed significantly elevated GFAP and Iba1 immunoreactivity in the striatum and substantia nigra pars compacta (SNpc) regions of the α-syn and MPTP mice group compared to the control group (Fig. 6G–I) and (Fig. 7D–F). Similarly, proinflammatory markers TNF-α, and IL-1β displayed enhanced expression in the brains of NSE-hαSyn mice (Fig. 6J–L). Notably, co-administration of Os_9aa with α-syn and MPTP substantially reduced the immunoreactivity of GFAP, Iba1, and TNF-α, and IL-1β in these regions in PD-induced mice.

### Os_9aa enhanced Nrf-2/HO-1 level by reducing oxidative stress in NSE-hαSyn transgenic mice brain

**Fig. 8:**
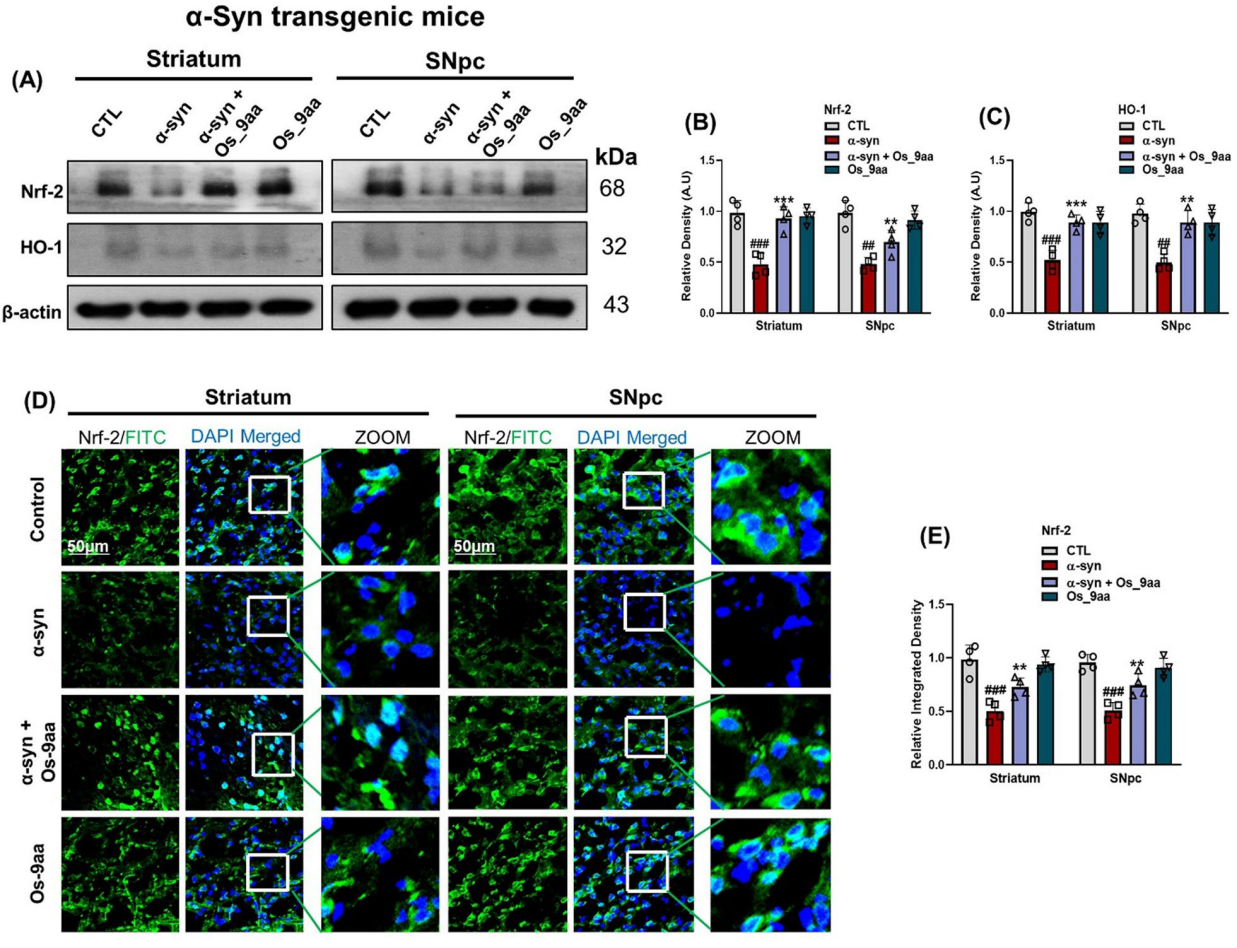
Os_9aa alleviated oxidative stress by upregulating the Nrf-2/HO-1 level. (**A**) Immunoblot analysis and corresponding histograms (**B**, **C**) for Nrf-2 and HO-1 in the striatum and SNpc of NSE-hαSyn transgenic mice brain respectively, using β-actin as a loading control. (**D**) Immunofluorescence images of Nrf-2, along with their respective histogram (**E**), in the striatum and SNpc of NSE-hαSyn mice. Scale bar = 50 µm, magnification 10 × . Graph values represent mean ± SEM with significant differences. *Indicates a significant difference between the control and α-syn groups; #indicates a difference between α-syn and Os_9aa-treated groups. Significance: #p ≤ 0.05, ##p ≤ 0.01, and ###p ≤ 0.001; *p ≤ 0.05, **p ≤ 0.01, and ***p ≤ 0.001

Nrf-2 and its downstream target, heme oxygenase 1 (HO-1), are pivotal in mitigating OS and preventing neurodegeneration. Dysregulation of the Nrf-2/HO-1 pathway leads to elevated OS, overproduction of ROS, and mitochondrial dysfunction [57]. Furthermore, we explored the protective antioxidative effects of Os_9aa against α-syn-induced OS in the NSE-hαSyn transgenic mice brain, mediated through the Nrf-2/HO-1 signaling pathway. In our western blot results, Os_9aa treatment significantly reduced the expressions of α-syn and Os_9aa + α-syn group of mice as compared control group and Os_9aa group of mice in the brain’s striatum and SNpc regions (Fig. 8A–C). Further, we also confirmed our results by immunofluorescence analysis. Remarkably, Os_9aa administration alleviated the α-syn expressions in the α-syn group and Os_9aa + α-syn group of mice, compared to the control group and Os_9aa group, specifically in brain’s striatum and SNpc regions (Fig. 8D, E). Overall, Os_9aa administration improved the level of OS in NSE-hαSyn PD transgenic mice brains.

## Discussion

**Fig. 9:**
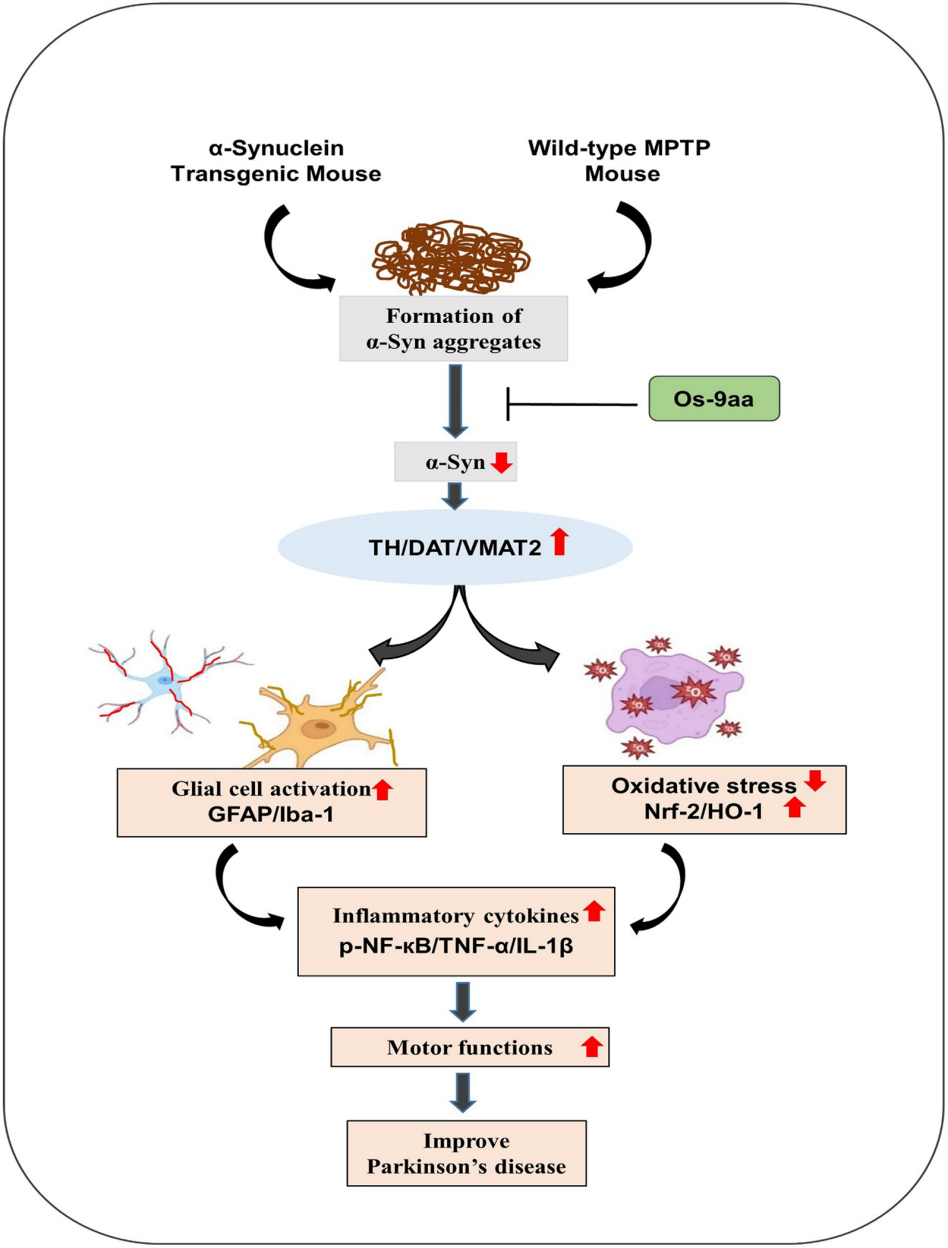
The neuroprotective approach of Os_9aa against NSE-hαSyn and MPTP PD mice brain

To begin with our experimental models, the transgenic NSE-hαSyn mice were used as an α-syn model while MPTP of 30 mg/kg (i.p) for 5 weeks as an MPTP model of PD. Currently, we reported and evaluated the neuroprotective properties of Os_9aa in transgenic NSE-hαSyn mice and MPTP-induced PD mice brains. Overall, our results demonstrated that the administration of Os_9aa improved cognitive and motor functions in the brains of NSE-hαSyn and MPTP mice groups. Os_9aa treatment improved cell viability, and mitigated cytotoxicity and caspase-3/7 activity in human neuroblastoma SH-SY5Y cells. Furthermore, Os_9aa administration significantly restored dopaminergic neurons and alleviated the level of OS, neuroinflammation, and neurodegeneration in NSE-hαSyn/MPTP-induced PD mice brains (Fig. 9).

First, we performed in vitro cell culture analysis using human neuroblastoma SH-SY5Y cells to examine the neuroprotective effects of Os_9aa on MPTP/α-syn-induced neurotoxicity. Consequently, we assessed cell viability, cytotoxicity, and caspase-3/7 activity (apoptosis) in a dose-dependent manner (Fig. 2A–C, and Fig. 2D–F, respectively). Our findings revealed that SH-SY5Y cells treated with Os_9aa restored and significantly upregulated the cell viability and reduced the cytotoxic cell death and apoptotic caspase-3/7 activity in MPTP/α-syn-induced PD mice brain (Fig. 2A–C, and Fig. 2D–F, respectively). Previous research studies also reported that MPTP/α-syn induces neuronal cytotoxicity, reduces cell viability, and causes apoptotic cascades in SH-SY5Y neuroblastoma cells [40, 58–61].

The cardinal feature of PD pathogenesis is the accumulation of high levels of presynaptic neuronal α-syn aggregates in the brain. In the PD-induced brain, SNpc encompasses a higher level of α-syn aggregates. However, these aggregates can also be found in other neurons located in the central nervous system (CNS) and peripheral nervous system (PNS) [62, 63]. Current research studies exhibited the potential positive effect of Os_9aa, which reversed behavioral alterations in NSE-hαSyn and MPTP PD mice models. Previous findings showed that α-syn and MPTP treatment significantly increased the expression of α-syn in the brains of experimental animals [16, 64, 65]. Our findings demonstrated that treatment with Os_9aa significantly reduced the overexpression of α-syn in NSE-hαSyn and MPTP mice brains. TH, VMAT2, and DAT serve as key markers of dopaminergic neurons, playing essential roles in dopamine synthesis and regulation in the brain. In PD, dysregulation of TH, VMAT2, and DAT occurs in the striatum and SNpc [66, 67]. Previous research has established that reductions in TH, VMAT2, and DAT are closely associated with dopamine depletion in the substantia nigra, a hallmark feature observed in PD patients. These deficits contribute to the progressive loss of dopaminergic neurons, major pathological characteristics of PD [68, 69]. Consistent with this understanding, our present study revealed a significant alleviation in TH, VMAT2, and DAT in NSE-hαSyn and MPTP mice brains. However, administration of Os_9aa significantly counteracted this effect, restoring the expression of these dopaminergic markers and signifying its potential in PD pathology.

Neuroinflammation is primarily driven by cellular immune components such as glial cells, including microglia and astrocytes within the CNS. Neuroinflammation is implicated in the pathogenesis of numerous neurological disorders, including Alzheimer’s disease, multiple sclerosis, traumatic brain injury, and PD [70, 71]. Previous studies demonstrated that different toxins trigger inflammatory cascades in the brain, the toxins α-syn and MPTP animal models exhibit activation of glial cells along with increased proinflammatory factors in the striatum and SNpc, which play a critical role in initiating neuroinflammation and subsequent neurodegeneration [5, 72–74]. Astrocytes and microglia maintain brain homeostasis and play a significant role in neuroprotection and regeneration of damaged brain cells [75, 76]. They are highly reactive to ROS and help to mitigate neuroinflammation. Nonetheless, an over-activation of astrocytes and microglia leads to the onset of neuroinflammatory processes [77]. In the current study, our western blot and immunofluorescence results showed higher expression levels of GFAP and Iba1 in the brain’s striatum and SNpc-regions-in response to α-syn and MPTP-induced mice groups. On the other hand, Os_9aa administration significantly mitigated the effect of α-syn and MPTP in mice groups of NSE-hαSyn and MPTP PD mice models. Along with this, in reactive gliosis, the activated glial cells and damaged neurons release inflammatory mediators such as p-NFκB, TNF-α, and IL-1β. These molecules are central to the initiation and progression of neuroinflammatory processes [78, 79]. Our findings revealed that these inflammatory biomarkers showed increased levels of expressions in brain regions striatum and SNpc in NSE-hαSyn and MPTP mice groups. Contrary, treatment of Os_9aa regulated and significantly alleviated the expressions of these inflammatory biomarkers in the striatum and SNpc region of the brain in NSE-hαSyn and MPTP mice groups.

OS and neuroinflammation are hallmark features of NDDs. OS plays a pivotal role in the degeneration of dopaminergic neurons as well as PD pathogenesis [80]. Nrf-2 is a transcription factor that regulates the expression of various protective genes, including HO-1, which shield cells against OS and neurodegeneration [50, 81]. Research has shown that peptide drugs have attracted the attention of researchers and biomedical industries because of their wide range of antibiotic and immunomodulatory activities [82]. Peptides, compared to other natural macromolecular compounds, possess the unique benefit of being non-toxic and free from side effects. To date, numerous peptides have been studied for their pharmacological properties, including their hypoglycemic, hepatoprotective, anti-inflammatory, and antioxidant activities [83–86]. In line with these studies, our findings revealed that Os_9aa treatment remarkably alleviated the level of OS by elevating the Nrf-2/HO-1expression level in NSE-hαSyn mice groups, suggesting the potent antioxidant effects of Os_9aa peptides against α-syn induced PD transgenic mice model (Fig. 8A–E).

## Conclusion

In conclusion, we developed Os_9aa, and investigated its neuroprotective properties in NSE-hαSyn and MPTP-induced PD mouse models. Os_9aa demonstrated significant antioxidative, anti-inflammatory, and neuroprotective effects, highlighting its potential as a therapeutic candidate for PD and related neurodegenerative disorders. This research study has limitations suggesting further studies involving mechanistic exploration, pharmacological characterization, long-term evaluation, and clinical validation are needed to confirm the therapeutic potential of Os_9aa in human PD.

## Data Availability

The manuscript contains all the necessary original data. Further inquiries can be directed to the corresponding author upon request.

## Abbreviations

Os_9aa: Osmotin-derived 9-amino-acid peptide
NSE-hαSyn: Neuron-specific enolase promoter human alpha-synuclein
NDDs: Neurodegenerative diseases
PD: Parkinson’s disease
α-syn: α-Synuclein
MPTP: 1-Methyl-4-phenyl-1,2,3,6-tetrahydropyridine
MPP +: 1-Methyl-4-phenylpyridinium ion
SNpc: Substancia Nigra pars compacta
i.c.v.: Intracerebroventricular
i.p.: Intraperitoneal
TH: Tyrosine hydroxylase
VMAT2: Vesicular monoamine transporter 2
DAT: Dopamine transporter
Iba-1: Ionized calcium-binding adaptor molecule 1
GFAP: Glial fibrillary acidic protein
p-NF-кB: Phosphorylated nuclear factor-κB
TNF-α: Tumor necrosis factor-α
IL-1β: Interleukin-1β
OS: Oxidative stress
Nrf-2: Nuclear factor erythroid-related factor 2
HO-1: Heme oxygenase 1
6-OHDA: 6-Hydroxydopamine
ATP: Adenosine triphosphate
IACUC: Institutional Animal Care and Use Committee
NaCl: Sodium chloride
DMEM: Dulbecco’s modified Eagle medium
PBS: Phosphate Buffer Saline
TRITC: Tetramethylrhodamine isothiocyanate
FITC: Fluorescein isothiocyanate
DAPI: 4′,6-Diamidino-2-phenylindole
S.E.M: Mean ± Standard Error Mean
ANOVA: Analysis of variance
β-actin: Beta-actin
